# Influenza B Lineages Have More in Common Than Meets the Eye. Trivalent Influenza Vaccines Trigger Heterotypic Antibodies Against Both Influenza B Viruses

**DOI:** 10.3389/fmicb.2021.737216

**Published:** 2021-11-11

**Authors:** Laura Sánchez-de Prada, Silvia Rojo-Rello, Marta Domínguez-Gil, Eduardo Tamayo-Gómez, Raúl Ortiz de Lejarazu-Leonardo, José María Eiros, Iván Sanz-Muñoz

**Affiliations:** ^1^Department of Microbiology, Hospital Clínico Universitario de Valladolid, Valladolid, Spain; ^2^National Influenza Center of Valladolid, Valladolid, Spain; ^3^Department of Microbiology, Hospital Universitario Río Hortega, Valladolid, Spain; ^4^Department of Anesthesia, Critical Care and Pain Medicine, Hospital Clínico Universitario de Valladolid, Valladolid, Spain

**Keywords:** influenza B, cross-immunity, age, elderly, sex, vaccines, adjuvants, mismatch

## Abstract

Influenza B is accountable for an important burden during flu epidemics, causing special impact in children and the elderly. Vaccination is the best approach to address influenza infections. However, one of the main problems of this virus is that two different lineages circulate together, Victoria and Yamagata; and trivalent vaccines, that only contain one of these lineages, are still in use. For that reason, if during an epidemic, the lineage not included in the vaccine predominates, a mismatch would occur, and the vaccine effectiveness will be very poor. In this work, we evaluated the cross-protection given by the trivalent Influenza vaccine and compared serological profiles based on age, sex, and the type of vaccine used. We performed a retrospective analysis of serum samples obtained before and after seasonal influenza vaccination during 20 seasons (1998–2018). The results showed that heterotypic reactivity between both influenza B lineages is common, but always lower than the homologous response. Age is a relevant factor for this cross-reactivity between both lineages, while the sex and the type of vaccine not. Vaccination with trivalent influenza vaccines elicits cross-reactive antibodies against both lineages, however, this response might not be enough to provide an appropriate serological protection in case of mismatch.

## Introduction

Influenza is an infectious respiratory disease caused in humans by Influenza viruses. It is typically characterized by seasonal epidemics that take place annually, but also sporadic and unpredictable global pandemic outbreaks every 10–50 years. Pandemics are caused by the introduction of a new influenza A virus strain antigenically different, usually of zoonotic origin, from the previous circulating. This lack of pre-existing immunity in humans is often associated with a higher severity of the infection and increased mortality (Krammer et al., 2018).

Prior to the emergence of two distinct phylogenetic lineages (B/Victoria and B/Yamagata) in the 1980s that currently co-circulate, the influenza B virus circulated as an homogeneous group and was first isolated in 1940 by Francis (1940) and Chen and Holmes (2008). In the 1980s, the B/Yamagata/16/88 lineage and its variants spread worldwide. Whereas, B/Victoria/2/87 lineage viruses remained geographically restricted to Asia during the 1990s, for reasons not entirely understood, to spread to the rest of the world in 2002 (Shaw et al., 2002; Jennings et al., 2018; Caini et al., 2019).

In contrast to Influenza A viruses, that have been profoundly studied, influenza B viruses have drawn not as much attention. The lack of a natural animal host (apart from seals), a slower mutation rate and not been responsible for pandemics, make them of less impact compared to influenza A viruses (Glezen et al., 2013). Nevertheless, the continuous co-circulation of influenza B viruses with Influenza A(H3N2) and A(H1N1) viruses during seasonal epidemics, and their contribution to morbidity and mortality have increased the interest on them (Van De Sandt et al., 2015).

Although there is general belief that influenza disease due to type B is milder than type A, studies show that both infections are clinically indistinguishable. Individuals of all ages can be affected; however, the incidence of complications is frequently higher in younger children and the elderly. Worthy of remark, higher rates of disease-associated complications and also deaths are seen in the elderly due to the presence of underlying chronic health conditions (Tafalla et al., 2016). Currently, influenza B infections are considered as more severe than A(H1N1) and less than those caused by A(H3N2). Although the disease affects all age groups, the incidence seems to be the highest among older children and young adults. Viruses of the B/Victoria lineage infect subjects at a relatively younger age than those of the B/Yamagata lineage (Van De Sandt et al., 2015). In addition, the incidence of influenza B infections can differ drastically from one influenza season to another (Ambrose and Levin, 2012). For those reasons, influenza B is an important cause of morbidity and mortality during inter-pandemic periods, and its prevention entails a global public health challenge (Jennings et al., 2018; Caini et al., 2019).

Several approaches are available to deal with seasonal influenza epidemics. Vaccination is considered the most effective option for preventing influenza disease as well as its complications. As influenza viruses undergo frequent changes in their surface antigens, new influenza vaccines are designed annually to match the circulating lineage expected for the next influenza season. Immunization programs worldwide commonly use the trivalent formulation, that could include an adjuvant or not, which contains viruses that represent 3 influenza strains – 2 influenza A strains [A(H1N1)pdm09 and A(H3N2) subtypes], and one influenza B lineage (B/Yamagata or B/Victoria) – recommended by the WHO. Epidemiological surveillance data show widespread co-circulation of these 2 B/lineages within the same influenza season since 2000. As one of these lineages is not covered by the annual vaccine, a mismatch with the circulating lineage could result in less effective vaccination campaigns and additional disease-related burden (Tafalla et al., 2016). Noticeably, in the North hemisphere, in the ten seasons between 2001 and 2011, the B lineage included in the vaccine only matched the predominant circulating lineage in half of the seasons. This is also frequent on the South hemisphere. In general, it has proved to be difficult to predict which influenza B virus strain will circulate in the coming season, and the cross-reactivity is limited in the case of a vaccine mismatch (Van De Sandt et al., 2015; Ortiz de Lejarazu et al., 2018).

The aim of this study is to evaluate the cross-protection given by the trivalent influenza vaccine and compare the serological effect of age, sex, and the use of adjuvanted versus non-adjuvanted influenza vaccine.

## Materials and Methods

### Study Design

A retrospective observational study was designed analyzing serum samples from a total of 3,446 healthy individuals recruited from the vaccination programs run by primary healthcare centers during the Influenza Vaccine Campaigns (IVCs) for 20 seasons (1998–2018). Pre-vaccination sera were obtained prior to the administration of the influenza vaccine and post-vaccination samples were obtained 28 days after vaccination to ensure a correct immunization. Serum samples are annually sent by the physicians of the Sentinel Surveillance Network of Castile and Leon (Spain) to the National Influenza Centre of Valladolid for their analysis as a part of the sero-epidemiological surveillance performed as a part of the WHO Global Influenza Surveillance and Response System (GISRS) regulated through the order SAN/1593/2006 of 13th of October 2006. Samples were stored at −20°C for correct transport and preservation. Informed consent was obtained, and the recruitment of patients was performed following Spanish Organic Law for Data Protection, patient’s rights and obligations for clinical documents (BOE n°298 of 14th December 1999, Law 41/2002). This research was performed according to the Declaration of Helsinki and was yearly approved by the Ethics Committee of East-Valladolid health area under the code PI 21-2314.

The administered seasonal trivalent influenza vaccines included the A and B influenza strains recommended by the World Health Organization (WHO) for the Northern hemisphere for each IVC. There were 11 seasons that included B/Yamagata lineage vaccine (BYv) and 9 seasons included B/Victoria lineage vaccine (BVv).

### Hemagglutination Inhibition Assay

Hemagglutination inhibition assay (HI) was performed to detect the presence of anti-hemagglutinant Abs in pre and post- vaccination serum samples. This analysis was conducted following the protocol recommended by WHO and the Influenza Surveillance Network for the surveillance of influenza viruses and vaccine efficacy (WHO, 2011). The serum samples had to be deactivated beforehand HI using RDE (Receptor Destroying Enzyme; Denka Seiken, Tokyo, Japan). This was performed by combining 100 μl of serum with 300 μl of RDE to remove non-specific inhibitors from the sera. This combination was incubated at 37°C in a water bath for 12–18 h and then inactivated for 1 h at 56°C. To perform HI, serial double dilutions of 50 μl of each serum were conducted in 96-V-microwell plates, and then 50 μl of a standard containing 4 hemagglutinin units (4HAU/25 μl) was incorporated to each well and incubated for 30 min at room temperature. Finally, 50 μl of hen erythrocytes at 0.75% were added and again incubated at room temperature for another 30 min. The Ab titer was defined as the highest dilution causing complete hemagglutination inhibition. The starting dilution is generally 1:10 and the lower limit of a detectable antibody titer is 10. When the titer of antisera is under a detectable threshold, due to a low or lack of antibodies, this is conventionally expressed as 5, half the lowest detection threshold (Trombetta et al., 2014). Pre and post vaccination titers were included in the database for their study.

### Statistical Analysis

The results were analyzed by using the classical serological parameters of the European Medicament Agency (EMA) for the evaluation of vaccine efficacy (The European Agency for the Evaluation of Medical Products, 1997; Petrie et al., 2015). Those include seroprotection rate (SPR), seroconversion rate (SCR) and geometric mean titer increase (GMTi) between post and pre-vaccination serum samples. Negative results in HI were assumed as half of the detection threshold (1/10). Seroprotection was considered when achieving an HI titer ≥ 1/40 and seroconversion was defined as a titer increase of at least four-fold between pre and post-vaccination sera. In addition, seroconversion was considered to have occurred in cases of negative pre-vaccination sera that achieved 1/40 titers after vaccination (Trombetta et al., 2014; Petrie et al., 2015). Different statistical parametric and non-parametric tests were used, using SPSSV27 (IBM, Armonk, NY, United States), and GraphPad Prism V9 (GraphPad, San Diego, CA, United States) and taking statistical significance at the *p* < 0.05 value.

## Results

### Population Characteristics

The mean age of all patients was 72.8 (IC95:72.3–73.3). A sum of 1,858 received a vaccine containing BYv and 1,588 received BVv. Mean ages were 73.1 (IC95%: 72.4–73.7) and 72.8 (IC95%: 71.8–73.3), respectively, and no significant differences were found between them (Student *T*-test, *p* = 0.291).

Population of study was divided then by age and sex (data collected since 2006). In addition, in the elderly, those analyses were performed contrasting by type of vaccine, Adjuvanted (AIV) and Non-Adjuvanted Influenza Vaccine (NAIV). The distribution of groups is detailed in Table 1.

**TABLE 1:** Description of the distribution of the different groups analyzed.

**N**	**B/YAMAGATA (BYv)**	**B/VICTORIA (BVv)**	***p*-value**
Male	402(42.6%)	564(58.4%)	0.28
Female	488(44.0%)	622(56.0%)	
15–64 years old	376(50.7%)	365(49.3%)	0.05
≥ 65 years old	1482(54.8%)	1223(45.2%)	
NAIV ≥ 65 years old	890(62.4%)	536(37.6%)	< 0.05*
AIV ≥ 65 years old	501(45.7%)	595(54.3%)	

Significant differences (*p* < 0.05) are marked with *.

### Humoral Status Before Vaccination

The serological status before vaccination of males and females who received BYv and BVv was similar against B/Yamagata lineage in terms of GMTs and SPR (Table 2). However, females showed significantly lower GMTs (91.4; IC95%:81.0–103.2) (Mann-Whitney, *p* < 0.05) as well as SPR (80.5%) (χ^2^, *p* < 0.05) against B/Victoria lineage in the cohort vaccinated with BYv. Nor was the case in the cohort vaccinated with BVv where no differences were found.

**TABLE 2:** Humoral status before vaccination against B/Yamagata lineage and B/Victoria lineage in all groups.

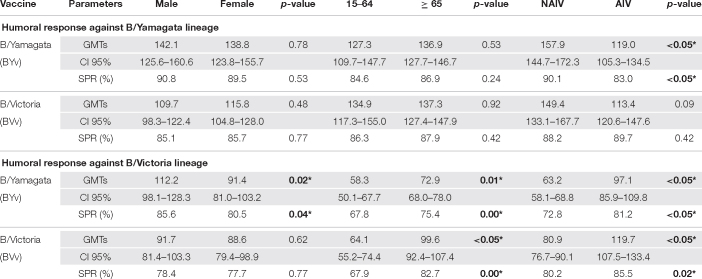

GMTs (Geometric Mean Titers), SPR (Seroprotection Rate). Significant differences (*p* < 0.05) are marked with *.

We analyzed differences between both age groups studied, young adults from 15 to 64 years and elderly ≥ 65. The serological status before vaccination against B/Yamagata lineage showed no significant differences in GMTs (Mann-Whitney, *p* < 0.05) and SPR (χ^2^, *p* < 0.05) when comparing both age groups, independently of the vaccine lineage received. On the other hand, the serological status against B/Victoria lineage showed significantly higher GMTs (Mann-Whitney, *p* < 0.05) and SPR (χ^2^, *p* < 0.05) in the elderly in both groups vaccinated either with BYv or BVv (Table 2).

We took into consideration the different vaccines received by population ≥ 65 because after 2005 the AIV was recommended for this age group, but the NAIV was being still used until then. Before vaccination, the serological status against B/Yamagata lineage was significantly higher in the group who received the trivalent BY-NAIV in terms of GMTs (Mann-Whitney, *p* < 0.05) and SPR (χ^2^, *p* < 0.05) but no differences were found in the group who received BVv. The serological pre-vaccination status against B/Victoria lineage showed significantly higher GMTs (Mann-Whitney, *p* < 0.05) and SPR (χ^2^, *p* < 0.05) in both groups who received trivalent BY-AIV and BV-AIV.

### Humoral Response to Vaccination

#### Humoral Response by Sex

Both BYv and BVv vaccines induced an homologous response against the strain contained in the vaccine as well as an heterologous response against the strain non-included. The homologous response to both vaccines was significantly higher in terms of GMTi (Mann-Whitney, *p* < 0.05), and SCR (χ^2^, *p* < 0.05) than the heterotypic response in both sexes (Table 3). The homologous responses triggered by both vaccines showed no differences in terms of post-vaccination GMTs, GMTi (Mann-Whitney, *p* < 0.05), SPR and SCR (χ^2^, *p* < 0.05) when comparing by the sex of the receptor. The comparison of the heterologous responses showed similar results and no differences were found in the previously mentioned parameters when comparing by the sex (Table 3).

**TABLE 3:** Comparison of the response to vaccination with BYv and BVv between males and females.

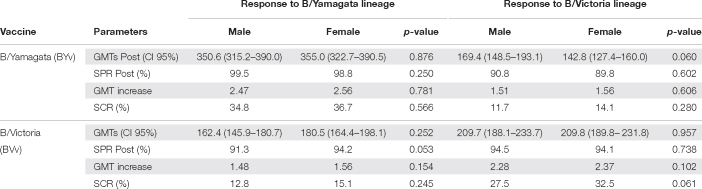

*GMTs (Geometric Mean Titers), SPR (Seroprotection Rate), SCR (Seroconversion Rate).*

#### Humoral Response by Age Group

**FIGURE 1:**
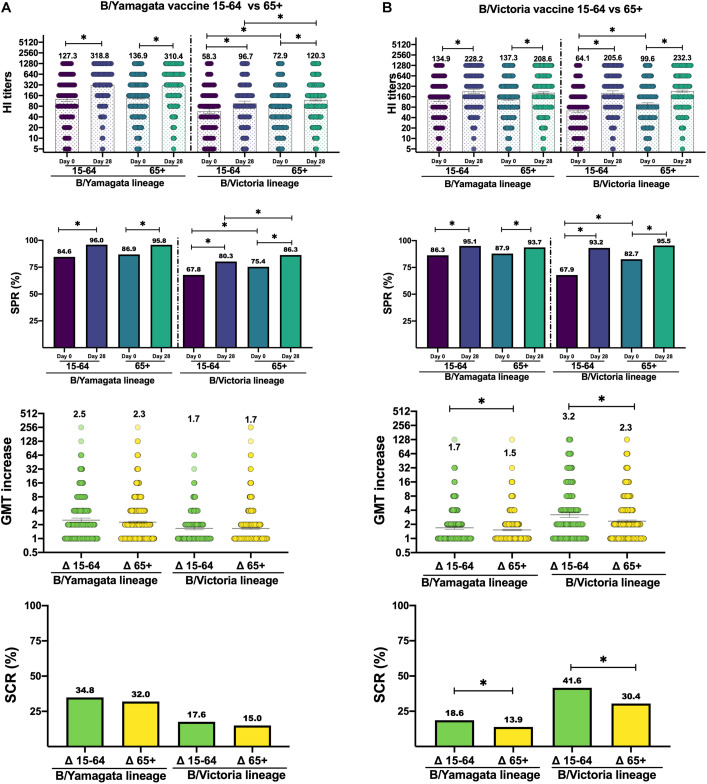
Values of hemagglutination titers, seroprotection rate (SPR), GMT increase (GMTi) and seroconversion rate (SCR). **(A)** Response to B/Yamagata vaccine in different age groups. **(B)** Response to B/Victoria vaccine in different age groups. Significant differences (*p* < 0.05) are marked with ^∗^.

Both BYv and BVv vaccines induced a significant homologous response in HI titers against the matching strain (contained in the vaccine), as well as a significant heterologous response against the strain non-included, regardless the age group of the receptor (Mann-Whitney, *p* < 0.05). The homologous response, against B/Yamagata lineage, to BYv showed no significant differences in post-vaccination GMTs, GMTi (Mann-Whitney, *p* < 0.05), SPR and SCR (χ^2^, *p* < 0.05) due to age, but the heterologous response, against B/Victoria lineage, produced significantly higher GMTs (120.3, CI95:112.7–128.4) as well as SPR (86.3%) (χ^2^, *p* < 0.05) in the elderly, while no differences were found in GMTi (1.66 vs. 1.65) (Mann-Whitney, *p* < 0.05) and SCR (17.6% vs. 15.0%) (χ^2^, *p* < 0.05) between both age groups (Figure 1). On the other hand, the homologous response, against B/Victoria lineage, to BVv produced similar results in both age groups in terms of GMTs (Mann-Whitney, *p* < 0.05) and SPR (χ^2^, p < 0.05), but a significantly higher GMTi (3.20 vs. 2.33) (Mann-Whitney, *p* < 0.05) and SCR (41.6% vs. 30.4%) (χ^2^, *p* < 0.05) was found in young adults. The heterologous response, against B/Yamagata lineage, to BVv showed significantly higher GMTi (1.69 vs. 1.52) (Mann-Whitney, *p* < 0.05) and SCR (18.6% vs. 13.9%) (χ^2^, *p* < 0.05) in adults (Figure 1).

#### Humoral Response by Type of Influenza Vaccine

**FIGURE 2:**
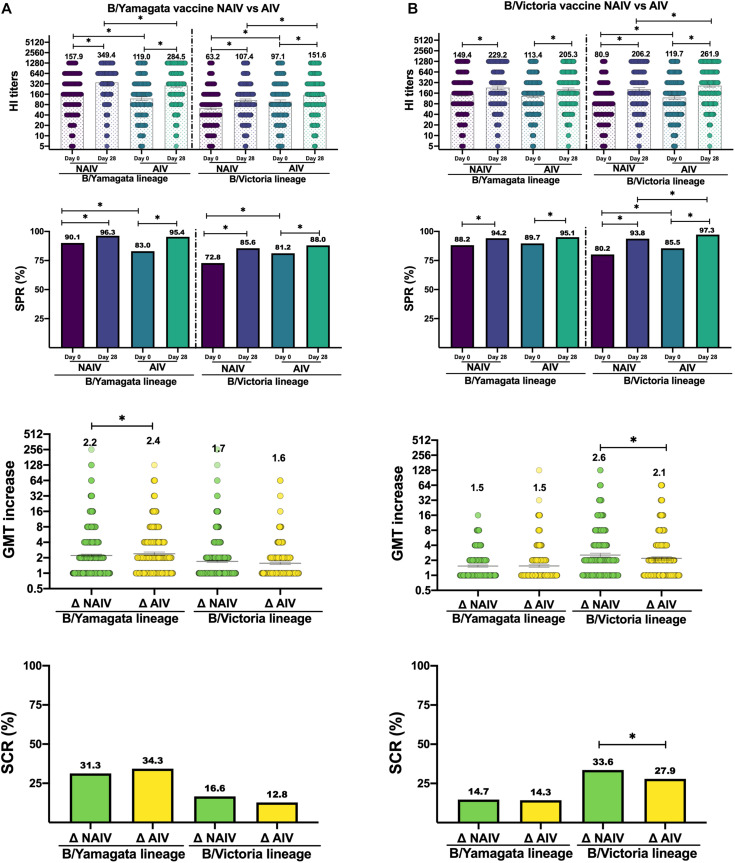
Values of hemagglutination titers, seroprotection rate (SPR), GMT increase (GMTi) and seroconversion rate (SCR). **(A)** Response to B/Yamagata vaccine by type of vaccine. **(B)** Response to B/Victoria vaccine by type of vaccine. Significant differences (*p* < 0.05) are marked with ^∗^.

The humoral response comparing by type of vaccine was only evaluated in the elderly. The homologous response, against B/Yamagata lineage, triggered by NAIV-BYv showed significantly higher post-vaccination GMTs (349.4, CI95%: 324.2–376.7) (Mann-Whitney, *p* < 0.05) than AIV-BYv. Despite this, significantly higher GMTi was observed when using the AIV-BYv (2.39 vs. 2.21) (Mann-Whitney, *p* < 0.05) (Figure 2). No differences were found between both types of vaccine in terms of SPR and SCR. The heterologous response, against B/Victoria lineage, triggered by AIV-BYv caused significantly higher post GMTs (151.6, CI95%: 134.9–170.4) compared to GMTs achieved with NAIV-BYv (107.4, CI95%: 99.0–116.5) (Mann-Whitney, *p* < 0.05). No differences between vaccines in SPR, GMTi and SCR were found (Figure 2).

The homologous response, against B/Victoria lineage, triggered by AIV-BVv induced significantly higher GMTS (261.9, CI95%:237.9–288.4) (Mann-Whitney, *p* < 0.05) as well as SPR (97%) (χ^2^, *p* < 0.05) and SCR (27.9%) (χ^2^, *p* < 0.05) than NAIV-BVv. However, the GMTi was significantly higher in those vaccinated with NAIV-BVv (2.55) (Mann-Whitney, *p* < 0.05). No significant differences were found in the heterologous response between both types of vaccine (Figure 2).

## Discussion

In contrast to influenza A viruses that can infect a wide range of animal hosts, influenza B infections are almost exclusively restricted to humans with only sporadic infections reported in wildlife (Osterhaus et al., 2000; Bodewes et al., 2013). However, in humans, influenza B accounts for 23% of the global of influenza infections, varying its frequency from season to season and also between different areas around the globe (Heikkinen et al., 2014; Caini et al., 2019). Most children older than eight years old have a basal immunity to influenza A, while comparable immunity against influenza B is only acquired at the age of 18 years old (Sauerbrei et al., 2014). Therefore, influenza B infection poses an important threat to infants presenting a disproportionate number of deaths compared to its circulation rates, reaching up to 52% of all influenza-related deaths (Glezen et al., 2013; Shang et al., 2018).

Our global analysis showed that, before vaccination, the humoral protection against the B/Victoria lineage was lower than the B/Yamagata lineage. This lower serological protection could be explained by different factors. First, B/Victoria is the eldest lineage and B/Yamagata lineage did not arise until the 1970s. Secondly, B/Victoria was restricted to Asia from 1992 to 2002 (Shaw et al., 2002), and since then, they have both co-circulated globally and alternating in regional dominance. Finally, B/Victoria lineage viruses appear to undergo more rapid lineage turnover and antigenic drift (Kanegae et al., 1990; Rota et al., 1990; Vijaykrishna et al., 2015; Langat et al., 2017). These three factors could have result in a lower humoral protection before vaccination due to a lesser contact with B/Victoria lineage, besides of a faster divergence that could lead to less presence of specific Abs.

As previous studies have shown, the serological response to vaccination lead to significant response toward both lineages, independently of which vaccine was used, BYv or BVv. This remarks the presence of cross-reactive Abs against the mismatched strain (Tricco et al., 2013; Skowronski et al., 2014; Beyer et al., 2017). Despite this, the response was always higher against the homologous strain than the heterologous strain, observing that GMTi is 36% and 26% higher than the heterotypic response triggered by BVv and BYv, respectively, and SCR is at least 2 times higher in response to both vaccines.

The comparison of the humoral status before vaccination between males and females showed that both sexes were similarly protected against both lineages, except for females that later received BYv. Females in this group showed slightly less humoral protection than men, but only against the B/Victoria lineage. Our results showed an absence of differences in the response to vaccination between both sexes, neither the against the matching nor the mismatching lineages. These findings are in contrast to influenza A viruses that induce higher responses in females after vaccination, and specifically in those whom are elderly (Couch et al., 2007; Engler et al., 2008; Furman et al., 2014; Sánchez-de-Prada et al., 2021).

On the contrary, the age seems to present an interesting and important role in protection against influenza B. Prior to vaccination, adults (15–64 years old) showed a lower humoral protection than the elderly. However, this may be not surprising since the elderly have been more frequently in contact with influenza B viruses due to their age. But this issue is interesting as we did not observe signs of immune senescence in the elderly for this type of virus. More striking is the significantly lower pre-vaccination protection displayed by both adults and elderly against B/Victoria lineage. In the last 20 years, the B/Yamagata has been predominant in 10 seasons, B/Victoria in six seasons and during four seasons they have co-circulated in nearly equal distribution. Also, it is important to recall the regional restriction of B/Victoria lineage to Asia for a decade until 2002 (Shaw et al., 2002). Therefore, the antigenic stimulus of the population seems to have been of a higher extent for B/Yamagata lineage compared to the exposure to B/Victoria lineage (European Centre for Disease Prevention and Control, 2021). All these factors, could explain why all patients exhibit such a lower protection against B/Victoria lineage.

For Influenza B viruses, the phenomena known as Original Antigenic Sin (OAS) and antigenic seniority have not been described. Different studies have detailed how B/Victoria lineages tend to infect very young children, while B/Yamagata lineage show a bimodal distribution with peaks in both children and adults over 25 years old (Caini et al., 2019; Virk et al., 2020). Original antigenic sin in this case would support a more intense response against B/Victoria if that’s what was first encountered in life. Our results suggest that BVv displays a higher degree of cross-reactivity compared to BYv, especially in adults. This contradicts previous studies that describe how antigenic drift of B/Victoria lineage, similar to that of A(H3N2), induces a limited cross-reactivity between phylogenetic clusters compared to B/Yamagata lineage, which resembles to A(H1N1) subtype, and has multiple variants in circulation, elicits greater levels of cross-reactivity (Vijaykrishna et al., 2015; Caini et al., 2019).

Interestingly, our results show how the response of adults to BVv triggers significantly higher homotypic, but also heterotypic responses to B/Victoria and B/Yamagata lineage, respectively, compared to the elderly. These findings are opposed to those found in a clinical trial conducted in infants under 2 years old, that showed a more intense serological response against B/Yamagata lineage compared to B/Victoria lineage after vaccination (Danuta et al., 2011). However, in our study the use of BYv does not induce different responses between both age groups in any of the lineages. Heterotypic responses have been previously described, but not specifically for one vaccine component and age group (Levandowski et al., 1991; van de Sandt et al., 2015; Sanz Muñoz et al., 2018).

Quadrivalent Influenza Vaccines (QIV) were introduced into WHO recommendations for yearly vaccine campaigns in 2013–2014 season together with the trivalent influenza vaccine (WHO, 2013). It was in the 2018/19 season, when QIV was recommended as first option for the first time by the WHO (WHO, 2018). Additionally, the European Centre for Disease Prevention and Control (ECDC) started the recommendation of QIV for influenza prevention from 2017 (European Centre for Disease Prevention and control, 2017). Our results support the use of QIV due to a larger coverage against both lineages, despite the heterotypic humoral responses found in our experiments. This is also supported by studies regarding the effect it would have in reducing the health and economic burden that actually supposes (Cré pey et al., 2020).

When we compared the humoral response depending on the type of vaccine (NAIV or AIV), our results showed that, despite the vaccine used, vaccination triggered homologous and heterologous Abs. The response to BYv showed higher homotypic response to AIV-BYv when compared to NAIV-BYv but no differences in the heterotypic response. Nevertheless, the response to BVv showed higher homotypic response with the NAIV-BVv despite higher total values of GMTs and SPR with the AIV-BVv. Despite these findings, we observed that the proportion of vaccines administrated for each lineage was not the same which could result in a bias and not entirely represent the actual differences. For that reason, the data presented in our study is not enough to extract a conclusion on whether adjuvants enhance responses in the case of B viruses. Further studies are needed with more homogeneous populations to determine the influence of adjuvants in B lineages and consider the importance of including them in the formulation of QIV.

One of the limitations of our study is that that antibody response was assessed by hemagglutination inhibition assay, which is considered the gold standard method to assess vaccine-induced antibody responses by the WHO (2011). This could result in information forfeited in terms of neutralizing Abs and cellular responses that could be influenced by factors such as age and sex. Another limitation is that we do not know if the participants were repeatedly vaccinated so we cannot determine the influence of this factor or the existence of negative interference for B viruses. On the other hand, as we only have data relative to age, sex and type of vaccine (in the case of the elderly), we could not perform a multivariate analysis. Finally, we have not analyzed in detail each season, which could result in the loss of particularities. However, a wide-ranging analysis of the data let us have a global picture of the humoral response against B viruses after influenza vaccination.

## Conclusion

Our study reflects that sex is not a relevant factor when it comes to humoral responses to influenza B viruses, although it must be taken in account when designing vaccination trials because it can influence the responses in many ways. However, the age is important to determine humoral response and should be acknowledged when designing vaccination strategies to establish whether to use adjuvants or high dose vaccines, as well as which groups to prioritize. When it comes to type of vaccines, the data available is not enough to establish the role of adjuvants in the humoral response to B viruses and should be studied in more detail. Finally, our study portrays that the simple act of vaccination with a trivalent influenza vaccine provides cross-reactive protection against the strain not contained in the vaccine. However, that cross-reactive response produced is not comparable to the protection provided against the matching strain contained in the vaccine which could result in loss of vaccine effectiveness in the case of mismatch. Therefore, Quadrivalent vaccines should be the next step in preventing influenza infection and used when available, especially in population at risk. But, in the case of vaccine shortage when the latter are not available, Trivalent vaccines could be useful to protect the population to a certain extent.

## Data Availability Statement

The raw data supporting the conclusions of this article will be made available by the authors, without undue reservation.

## Ethics Statement

The studies involving human participants were reviewed and approved by Ethics Committee of East-Valladolid health area. Written informed consent to participate in this study was provided by the participants’ legal guardian/next of kin.

## Author Contributions

SR-R, MD-G, JE, and ROL-L designed the methodology of the study. IS-M, SR-R, ROL-L, and JE supervised the laboratory work. LS-D performed the formal analysis supervised by IS-M and JE and prepared the figures. LS-D, ROL-L, and IS-M prepared the original draft. JE and IS-M revised and edited the final manuscript. All authors have read and agreed to the published version of the manuscript and contributed to the preparation of the manuscript.

## Conflict of Interest

The authors declare that the research was conducted in the absence of any commercial or financial relationships that could be construed as a potential conflict of interest.

## Publisher’s Note

All claims expressed in this article are solely those of the authors and do not necessarily represent those of their affiliated organizations, or those of the publisher, the editors and the reviewers. Any product that may be evaluated in this article, or claim that may be made by its manufacturer, is not guaranteed or endorsed by the publisher.
